# Assay Performance and Quantification Limits in Perioperative Serum Cytokine Measurement After Total Hip Arthroplasty: A Secondary Laboratory Analysis

**DOI:** 10.3390/jcm15207829

**Published:** 2026-10-10

**Authors:** Arabela-Codruța Cocea (Anastasescu), Dan-Cristian Munteanu, Mădălina Popa, Cristian Ioan Stoica

**Affiliations:** 1 Doctoral School, “Carol Davila” University of Medicine and Pharmacy, 050474 Bucharest, Romania; cristian.stoica@umfcd.ro; 2 Faculty of Medicine, “Carol Davila” University of Medicine and Pharmacy, 050474 Bucharest, Romania; dan-cristian.munteanu@drd.umfcd.ro; 3 Infectious Diseases Department–Hemorrhagic Fevers, National Institute for Infectious Diseases “Prof. Dr. Matei Balș”, 021105 Bucharest, Romania; 4 Immunology Laboratory, National Institute for Infectious Diseases “Prof. Dr. Matei Balș”, 021105 Bucharest, Romania; madaspopa@yahoo.com

**Keywords:** total hip arthroplasty, interleukin-6, interleukin-10, perioperative inflammation, cytokine kinetics, surgical stress response, enzyme-linked immunosorbent assay, assay performance, limit of quantification, left-censored data, optical density, data recovery

## Abstract

**Background**: Commercial cytokine assays may not span the concentrations encountered in perioperative serum. We examined the extent to which reconstructed enzyme-linked immunosorbent assay data from a total hip arthroplasty cohort could support temporal biological interpretation when most IL-6 and IL-10 results were below the validated quantification range. **Methods**: This post hoc secondary laboratory analysis used serum collected preoperatively and at 6, 24, and 48 h later from 44 of 52 participants in a single-centre three-arm trial. Five cytokines were measured by sandwich ELISA. Optical-density (OD) values were reconstructed from scanned reports and triangulated across three transcriptions. OD trajectories were summarised with within-patient rank tests as exploratory assay-signal observations. Five omnibus tests were Holm-adjusted across analytes. Detectability was analysed as a paired binary outcome using Cochran’s Q and exact McNemar comparisons with Holm correction. Clinical characteristics, acute-phase markers, and missing-data structure were evaluated in the assayed subcohort. **Results**: The assayed subcohort included all 18 no-block participants and 26 of 34 block recipients; the 8 non-assayed participants were late-enrolled block recipients. IL-6 OD varied over time (Friedman raw *p* = 2.6 × 10^−11^; Holm-adjusted *p* = 1.3 × 10^−10^), but 154/176 readings (87.5%) were below the LLOQ. IL-6 detectability was 0/44, 9/44, 7/44, and 6/44 at the four timepoints (Cochran Q = 12.27, *p* = 0.0065); only the preoperative-to-6 h McNemar comparison remained significant after correction (adjusted *p* = 0.023). TNF-alpha, IL-1alpha, and IL-10 OD also varied in exploratory analyses, whereas IL-1beta was inconclusive. CRP rose from a median 0.20 mg/dL preoperatively to 7.70 mg/dL at 24 h and 14.55 mg/dL at 48 h in the same 44 participants. **Conclusions**: The data document an assay-range and data-recovery problem more securely than they establish cytokine kinetics. The reconstructed OD changed systematically after surgery, especially for IL-6, but measurements below the validated range cannot establish concentration ordering, cross-cytokine dominance, or counter-regulation. Because re-assaying was not possible, all biological interpretations are presented as tentative and non-transferable. The reproducible findings are the extent of censoring, the limits of the available assay records, and the need to match the assay range to expected perioperative concentrations before clinical sampling.

## 1. Introduction

Total hip arthroplasty induces a reproducible systemic inflammatory response to surgical tissue injury [1]. Its intensity and timing carry clinical weight, since the perioperative cytokine milieu is linked to postoperative fever, to the early recovery trajectory, and to the biology that multimodal and regional analgesic strategies are presumed to modify [2,3,4,5,6]. Serum cytokines have been studied as markers of this response, but their interpretation depends on sampling time, pre-analytical handling and whether the assay’s calibrated range covers the concentrations generated by the procedure. Values outside that range cannot be converted into biologically interpretable concentrations merely by applying more elaborate statistical methods.

IL-6 is the dominant systemic signal. In patients undergoing elective operations of graded severity, Cruickshank and colleagues showed that serum IL-6 rises within 2–4 h of incision in every patient, that the magnitude of the rise tracks the extent of the surgical injury (r = 0.80 with the duration of the surgery, *p* < 0.001), and that it correlates with and precedes the C-reactive protein (CRP) response (r = 0.67, *p* < 0.001) [7]. Subsequent work catalogued the determinants of the postoperative serum IL-6 concentration and its wide interindividual spread [8]. In joint replacement specifically, IL-6 has been proposed as a practical inflammatory marker [9], its time-course after elective THA has been described directly [10], and contemporary cohorts have refined the picture, under enhanced-recovery care [11] and across the first postoperative week [12]. After hip and knee arthroplasty, the reported circulating IL-6 maxima range from tens to several hundred pg/mL, with peak times between about 6 and 48 h depending on the procedure, sampling schedule, perioperative pathway, and assay [9,10,11,12,13,14]. Preoperative concentrations are typically a few pg/mL [11].

What that literature does not supply is a settled peak time. Wirtz and colleagues, sampling 30 patients after 20 hip and 10 knee replacements, report a peak of 399 ± 140 pg/mL at 6 h with a mean half-life of 15 h [9]. Minetto and colleagues, in 21 patients receiving an uncemented hip prosthesis, report a median peak of 82.3 pg/mL, with the rise correlated to patient age and its slope to the duration of the surgery [10]. Maniar and colleagues, sampling preoperatively and at 12 h, 48 h, four days, and two weeks after knee arthroplasty, place the IL-6 maximum at 12 h [14], while Huang and colleagues, sampling at 12 h, 48 h, 72 h, and two weeks under an enhanced-recovery pathway, found near-identical concentrations at 12 h and 48 h (54.48 ± 77.87 and 56.29 ± 41.20 pg/mL) with the nominal maximum at 48 h and no sample in between [11]. A recent synthesis places the peak “often within the first 6–24 h” [13]. The procedure, sampling schedule, perioperative pathway, and assay platform each move the answer, and no series in this literature samples densely enough between 6 and 24 h to locate the peak with confidence.

The cytokines upstream of IL-6 behave far less predictably in the systemic circulation, and the compartment in which they are measured matters. Sampling drained the wound blood and systemic blood in parallel after major orthopaedic surgery; Krohn and colleagues found that IL-1β and IL-6 rose significantly in drained blood while TNF-α rose only in arterial blood, and that IL-10 rose in both [15]. Elective orthopaedic surgery used as a controlled human model gives a similarly modest and inconsistent systemic IL-1 and TNF-α response relative to IL-6 [16,17]. The same asymmetry appears in hip replacement specifically: in a multiplex survey of 30 serum cytokines in 20 patients undergoing total hip replacement, IL-6 rose significantly at 6 h and at one day, whereas TNF-α and IL-1β showed no significant change at any postoperative timepoint [18]. More recently, serum and synovial fluid sampled in parallel around hip and knee arthroplasty showed IL-6 elevated in synovial fluid rather than in serum, while serum IL-10 was the analyte that rose consistently after surgery—a rise driven by the hip arthroplasty patients [19]. Opposing the pro-inflammatory arm, IL-10 is the principal counter-regulatory mediator, and the quantity of clinical interest is the balance between hyperinflammation and immunosuppression rather than either limb alone [20].

The parent cohort did not receive NSAIDs, COX-2-selective inhibitors, dexamethasone or gabapentinoids. However, participants differed in regional block exposure, ropivacaine dose, pain, and rescue analgesia. The cohort is therefore free of specified anti-inflammatory co-medication, not pharmacologically unmodified. These exposures and the non-proportional composition of the assayed subcohort must be considered when describing pooled temporal observations.

The principal motivation for this report is methodological. The original digital instrument export was unavailable, most IL-6 and IL-10 results were below the LLOQ, and portions of two IL-1 plates were structurally unusable. The available evidence can therefore describe the recovered assay signal, censoring, and data-loss mechanism, but it cannot establish validated concentration kinetics.

This post hoc secondary analysis had three objectives: to quantify censoring and data completeness; to describe exploratory within-patient OD changes without treating them as validated concentration trajectories; and to evaluate what the recovered records reveal about assay fitness for perioperative serum. Cross-cytokine dominance, counter-regulation, the and absence of an IL-1 response were not treated as testable conclusions.

## 2. Materials and Methods

### 2.1. Design, Setting, and Participants

This is a laboratory sub-study of a single-centre, three-arm controlled trial of the ultrasound-guided supra-inguinal fascia iliaca compartment block in primary uncemented total hip arthroplasty, conducted at the Foișor Clinical Hospital of Orthopaedics, Traumatology and Osteoarticular Tuberculosis, Bucharest, Romania. Enrolment ran from September 2024 to April 2025.

Patients were eligible if they were of American Society of Anesthesiologists (ASA) physical status II–III, aged 50–80 years, scheduled for an uncemented total hip arthroplasty, with a body mass index of 20–32 kg/m^2^. Exclusion criteria were contraindication to spinal anaesthesia; coagulopathy; renal or hepatic dysfunction; allergy to any study drug; NSAID use within the preceding five days; diabetes mellitus, rheumatoid arthritis, psoriasis, ankylosing spondylitis, peripheral neuropathy, or Parkinson disease; neoplastic disease; glucocorticoid therapy; opioid dependence; aorto-femoral bypass; and inability to understand the study or unwillingness to participate.

The parent trial was a single-blind, pragmatic, three-arm randomised controlled trial in patients with hip osteoarthritis and an indication for primary uncemented total hip arthroplasty through a lateral approach. Three parallel arms were formed: arm 1, no block (*n* = 18); arm 2, supra-inguinal fascia iliaca compartment block (S-FICB) with 40 mL ropivacaine 0.2% (*n* = 18); and arm 3, S-FICB with 40 mL ropivacaine 0.25% (*n* = 16). Participant flow is shown in Figure 1 No sham was performed; therefore, participants were not formally partial blinded to the procedure. The clinical personnel who recorded pain scores and titrated rescue analgesia were blinded to allocation. The laboratory personnel and the analyst were blinded to arm identity.

Participants were allocated by restricted randomisation using variable-length allocation series: consecutive participants were assigned to the same arm in series of 1, 2, 3, 4, or 6 participants, with both the series length and the order of the arms determined manually from a table of random numbers. Variable series length was used to reduce predictability of the allocation sequence. The intended allocation ratio was 1:1:1. Because allocation was applied in per-arm series rather than within fixed balanced blocks, arm totals were not constrained to be equal at interim points; the maximum cumulative between-arm imbalance during recruitment was 10 participants, and the final allocation was 18:18:16. The realised allocation sequence and the cumulative arm balance across enrolment are shown in Figure 1, and the full allocation schedule is provided as Figure 2. Allocation concealment relied only on the variable series length; no sealed-envelope or central (telephone- or web-based) allocation mechanism was used, so concealment was partial, and selection bias in arm allocation cannot be excluded; this mainly affects the arm-stratified summaries (Table S2). One participant randomised to the 0.2% arm (P3) had the administered concentration not recorded in the source documents; consistent with the intention-to-treat principle, this participant was analysed as randomised (0.2% arm).

Allocation proceeded in consecutive same-arm series of variable length, and samples were plated in patient-number order. Consequently, trial arm, enrolment order, storage interval, and plate membership may be related. This is relevant to the pooled description and especially to analytes restricted to one plate; no claim is made that pooling removes this source of heterogeneity.

Cytokines were assayed in 44 of the 52 participants (participants 1–42, 44, and 49); participant numbers followed enrolment order. The eight participants without cytokine data (43, 45–48, and 50–52) were not assayed, for laboratory logistical reasons, and all were late-enrolled block recipients. The assayed subcohort therefore includes all 18 no-block participants but only 26 of 34 block recipients and is not a random or proportionally representative sample of the parent trial. Because allocation proceeded in same-arm series and samples were assayed in enrolment order, trial arm, enrolment period, and plate membership are related.

### 2.2. Anaesthesia, Surgery, and Perioperative Analgesia

All patients were premedicated with 1–2 mg intravenous midazolam and received spinal anaesthesia at the L3–L4 interspace with 15 mg hyperbaric bupivacaine. No intrathecal opioid was given in any arm. Monitoring comprised electrocardiography, non-invasive arterial pressure, and pulse oximetry. Surgery was a primary uncemented total hip arthroplasty through a lateral approach.

Postoperative analgesia was threshold-triggered rather than scheduled: paracetamol at a pain score of 4 and morphine at a score of 7, with nefopam available on the same principle. No NSAID, COX-2-selective inhibitor, dexamethasone, metamizole, or gabapentinoid was administered at any point in any arm. This is a material feature of the cohort for present purposes. Each of these agents modifies the acute-phase cytokine response, and their complete absence—reinforced by recent NSAID use being an exclusion criterion—means the profile described here has not been pharmacologically damped. Regional block, ropivacaine, and rescue analgesia nevertheless differed between participants and are reported as perioperative exposures.

### 2.3. Blood Sampling and Cytokine Assays

Four blood samples were obtained from each participant: a baseline sample (A), drawn before antibiotic prophylaxis was given, and samples drawn 6 h (B), 24 h (C), and 48 h (D) after the baseline sample. Intervals were therefore counted from the baseline sample rather than from the end of surgery; with a median operative duration of 90 min, sample B was obtained before the sixth postoperative hour.

Samples were processed immediately after collection. Serum was separated by centrifugation at 5000 rpm for 5 min, aliquoted (2 mL per sample), transferred into 2 mL cryotubes, and stored at −80 °C until analysis, without any freeze–thaw cycle before the assay. All samples were assayed on 25 May 2026, so storage time from collection to assay ranged from 410 to 614 days (median 567 days; 13.5–20.2 months); the four samples of each participant were collected within 2 days, so their storage times differed by at most 2 days.

Five cytokines were measured by sandwich enzyme-linked immunosorbent assay (ELISA) using FineTest kits (Wuhan Fine Biotech, Wuhan, China): IL-6, TNF-α, IL-1α, the high-sensitivity variant of IL-1β, and IL-10 (Table 1). For each analyte, the two plates came from the same kit lot; lot numbers were not available. Each 96-well plate contained a seven-point standard curve (serial 1:2 dilutions) and a blank in its first column and 88 samples (22 participants × four timepoints) in the remaining wells, so samples and calibrators were assayed in singlicate and no additional control samples were included. Participants were assigned to plates in enrolment order (plate 1, participants 1–22; plate 2, participants 23–42, 44, and 49), with all four samples from a participant on the same plate. Storage time was therefore almost completely separated by plate (plate 1, 565–614 days, median 596; plate 2, 410–567 days, median 482), whereas it was similar in the no-block and block groups (medians 581 and 562 days). Sample positions were not randomised, so timepoint was linked to plate position. Laboratory personnel were blinded to treatment allocation and sampling timepoint.

Absorbance was read at 450 nm on a BIOMED LEDETECT 96 reader with MikroWin (2020 Update, Version V6.22), which derived concentrations by four-parameter logistic (4PL) regression and flagged results outside the reportable range. OD values were analysed as printed. Intra- and inter-assay coefficients of variation were not available, and plate- and result-acceptance criteria were not documented in the available records.

For IL-1α, the OD values and standard-curve record of the second plate could not be recovered from the scans, although its printed result flags were legible; for IL-1β, the first plate was corrupt. Plate membership therefore determined the population analysable for OD for these two analytes.

### 2.4. Data Recovery and Triangulation Cross-Validation

Results were supplied as the plate reader’s printed reports, scanned as image-only PDFs without a text layer. Optical density was cross-validated by triangulating three independent representations of the same scanned plate reports: the reports re-parsed from their Office-format transcription; a prior OCR-structured extraction; and an independent human transcription performed from 300-DPI scans, which also recorded per-cell below-limit (BLQ) and above-limit (ALQ) flags. Row identity and plate alignment were anchored by requiring each grid row’s standard-curve value to match the independently known quality-control standard-curve optical density for that row (±0.02). Agreement was assessed both strictly and in an order-independent, row-anchored manner immune to off-by-one column slips.

Two complementary comparisons were made, over different sets of cells. First, corroboration: of the 168 optical-density cells for which a cross-check was possible, 158 (94%) were confirmed by at least two independent sources, while, for the remaining 10, no legible counterpart existed in the matching plate-report row. Second, a stricter cell-by-cell comparison over the 138 cells carrying a value in both sources initially flagged 32 mismatches; inspection showed 29 of these to be optical-character-recognition column-alignment artefacts, in which the same value reappears in the neighbouring cell, leaving 3 genuine value conflicts between sources (strict agreement 135 of 138). Because the two comparisons run over different sets of cells, the three conflicts are not a subset of the 168 and the two counts should not be added. Cells back-filled with a patient-identifier integer, or lying outside a plausible optical-density range, were reclassified as illegible and treated as missing.

Triangulation establishes reproducibility of transcription from the surviving printouts, not analytical validity or biological accuracy. All sources ultimately derive from the same scanned reports, and no digital MikroWin export was available.

One region of the source material carries lower confidence than the rest and is identified here rather than left implicit. On the IL-1α second plate, the scan was slightly tilted, and the row corresponding to timepoint A in the even-numbered patients required column alignment to be inferred from the pattern of the remaining timepoints in the same patients. Those cells contribute to the detectability tabulation but not to the rank analysis, from which the plate is excluded in its entirety.

All analyses were computed on the single reconciled optical-density variable produced by this procedure. It agrees with the prior OCR reading in 622 of the 634 jointly populated cells, resolves the remaining 12 cross-source discrepancies in favour of the reading corroborated by the plate-report source, and incorporates 21 values recoverable only from that source. The reconciled dataset therefore contains 655 populated cells of 880 (74.4%). No result in this paper rests on any single transcription.

Below-limit and above-limit flags were transcribed from the printed concentration fields and were available for all 176 patient-timepoint cells per analyte, including cells whose OD digits were illegible. Detectability is therefore analysable in all 44 assayed participants, whereas OD rank analyses use analyte-specific complete cases.

### 2.5. Statistical Analysis

All cytokine analyses were post hoc and exploratory. For each analyte, OD was summarised in patients with all four timepoints and compared with a Friedman test followed by Holm-adjusted Wilcoxon signed-rank contrasts. The five analyte-level omnibus *p*-values were additionally adjusted by the Holm method. Medians and interquartile ranges are emphasised; statistical significance is not treated as validation of concentration ordering below the calibrated assay range.

Detectability was defined from the printed concentration field as a numeric result or a result flagged above the ULOQ (ALQ), versus a result flagged below the LLOQ (BLQ). Because the same participants were measured repeatedly, the four timepoints were compared with Cochran’s Q. When the omnibus test was significant, exact paired McNemar comparisons were reported with Holm correction across the six pairs. Detectability flags were available for all 44 assayed participants at all timepoints; no imputation was used.

Clinical characteristics were summarised for the 44 assayed and 8 non-assayed participants. Arm-stratified OD summaries and a no-block sensitivity analysis were descriptive and did not test treatment effects. The previous comparison restricted to quantifiable concentrations was removed because conditioning on post-treatment detectability can introduce selection bias.

OD is plate- and instrument-specific. Within the calibrated range, it may preserve concentration order, but monotonicity was not established below the lowest calibrator and is degraded at the upper plateau. Accordingly, the OD analyses describe assay signal rather than validated cytokine concentration kinetics.

The IL-6 curve also flattened above approximately 37 pg/mL, reducing discrimination among the few high signals. At the opposite end, most IL-6 and IL-10 measurements were below the lowest calibrator. These two boundaries preclude reliable effect sizes in physical units and comparisons of magnitude across analytes.

No primary cytokine was prespecified. The analysis was developed after inspection of the assay records; all five analytes, pairwise contrasts, and sensitivity analyses are therefore labelled exploratory.

Analyses used two-sided alpha = 0.05. Computations were performed on the reconciled long-format dataset.

Because the panel is heavily left-censored, the proportion of readings below the limit of quantification was tabulated per cytokine and per timepoint and treated as informative in its own right, following the detectability framing of Ahmadi and colleagues [21]. Throughout, a reading is counted as detectable when the instrument returned a numeric concentration rather than a below-limit flag; readings above the upper limit of quantification are therefore detectable but not quantifiable. Detectability can be analysed without trusting the absolute concentration, and it was compared across timepoints with Cochran’s Q test and paired exact McNemar comparisons, as described above. Rank methods degrade gracefully when many low readings are tied near the floor, but they lose power precisely where censoring is heaviest. Tobit-type regression for censored correlated concentrations [22] and two-part or detectability models [21,23] are the appropriate treatments once validated concentrations are available; the choice of rank-based inference here follows the endorsement of distribution-free methods for non-normal censored immunological data [24].

### 2.6. Ethics

Our study was approved by the Ethics Commission of the Foișor Clinical Hospital of Orthopaedics, Traumatology and Osteoarticular Tuberculosis (approval no. 6777, 10 July 2023) and by the Ethics Council of the Carol Davila Faculty of Medicine (approval no. 11581, 1 April 2024), and was conducted in accordance with the Declaration of Helsinki. Written informed consent was obtained from every participant. The parent trial was not prospectively registered. No public protocol or statistical analysis plan specific to this post hoc cytokine analysis is available.

## 3. Results

### 3.1. Study Population and Data Structure

The assayed subcohort comprised 44 participants (18 no block, 26 block). Compared with the eight non-assayed participants, it included fewer women and fewer ASA III participants, it had a lower median body mass index, longer operations, and higher rescue analgesic use, and all non-assayed participants had received a block (Table 2). The laboratory subcohort is therefore a selected rather than a random sample of the parent trial.

Values are median [IQR] or *n* (%). Standardised difference: Assayed minus non-assayed, as the difference in means (continuous variables) or proportions (categorical variables) is divided by the pooled standard deviation; descriptive only, no hypothesis tests. Total blood loss is the sum of intraoperative, 24 h, and 48 h records. All eight are non-assayed.

The recovered cytokine grid contained 880 patient-timepoint-analyte cells. Detectability flags were available for all cells, whereas complete OD trajectories differed by analyte because of scan quality and whole-plate loss (Figure 3).

The IL-1alpha OD analysis was restricted to 21 plate-1 patients; the second plate could not be recovered. Because plate membership followed enrolment order, this result cannot distinguish a temporal OD pattern from plate, storage, or secular effects.

IL-1beta OD analysis was restricted to 16 plate-2 patients after the exclusion of the corrupted first plate. Its non-significant result is inconclusive, not evidence that systemic IL-1beta did not change.

Complete four-timepoint OD data were available for 39 IL-6, 32 TNF-alpha, and 33 IL-10 participants. The clinical characteristics of each complete-case subset are given in Supplementary Table S1.

Missingness was not plausibly missing completely at random. It arose from the scan quality, plate failure, and non-random omission of late-enrolled block recipients; the direction and magnitude of the resulting bias cannot be corrected from the surviving records.

### 3.2. Exploratory Optical-Density Summaries

Exploratory OD summaries are presented in Table 3 and Figure 3. After Holm adjustment across the five analyte-level omnibus tests, IL-6, TNF-alpha, IL-1alpha, and IL-10 remained statistically significant; IL-1beta did not. These *p*-values identify systematic OD variation but do not validate concentration trajectories outside the calibrated range. The IL-1α result is confined to plate 1, so a temporal pattern cannot be separated from plate or enrolment-period effects.

The median OD was at 450 nm with the interquartile range at four recorded timepoints. The IL-6 panel uses a truncated vertical axis beginning above zero. The OD values are the assay signal, not validated cytokine concentrations. OD values below the calibrated range may not preserve the biological concentration ordering; panels must not be compared in magnitude across cytokines.

IL-6 OD varied over time (raw Friedman *p* = 2.6 × 10^−11^; Holm-adjusted across analytes *p* = 1.3 × 10^−10^; *n* = 39). The nominal OD maximum occurred at 24 h, but 6 h and 24 h did not differ significantly, and the heavy BLQ fraction prevents this pattern from establishing an IL-6 concentration maximum.

TNF-alpha OD varied over time (raw *p* = 5.8 × 10^−4^; Holm-adjusted *p* = 0.0023; *n* = 32), with only the 24–48 h contrast surviving the within-analyte correction. The amplitude cannot be compared with other cytokines.

IL-10 OD varied over time (raw *p* = 6.6 × 10^−4^; Holm-adjusted *p* = 0.0023; *n* = 33), with the 24–48 h contrast being significant. Temporal concurrence with IL-6 does not demonstrate counter-regulation, and no mediator relationship is inferred.

IL-1alpha OD varied in the 21 plate-1 patients (raw *p* = 0.0010; Holm-adjusted *p* = 0.0023), but the single-plate restriction, enrolment-order relation, and unknown storage duration make a biological decline uncertain.

IL-1beta was not significant (*p* = 0.196; *n* = 16, plate 2 only). The result is described as inconclusive because the structural data loss, censoring, and small sample size limit both power and generalisability.

The recovered optical density by timepoint are presented in Figure 4.

### 3.3. Detectability and Left-Censoring

Of the 880 results, 585 (66.5%) were flagged below range, 262 (29.8%) were numeric, and 33 (3.8%) were flagged above range (Table 4). The below-range proportion was 87.5% for IL-6, 80.7% for IL-10, 63.1% for IL-1α, 54.0% for TNF-α, and 47.2% for IL-1β; all above-range results occurred for IL-1α (12) and IL-1β (21).

Detectability by timepoint is shown in Table 5 and Figure 5. After Holm adjustment across analytes, detectability changed across timepoints for IL-6 (Cochran’s *Q* = 12.27; *p* = 0.0065; adjusted *p* = 0.027) and TNF-α (*Q* = 12.67; *p* = 0.0054; adjusted *p* = 0.027), but not for IL-1α (*Q* = 8.27; *p* = 0.041; adjusted *p* = 0.12), IL-10 (*Q* = 7.89; *p* = 0.048; adjusted *p* = 0.12), or IL-1β (*Q* = 3.00; *p* = 0.39; adjusted *p* = 0.39).

IL-6 detectability was 0/44 preoperatively, 9/44 at 6 h, 7/44 at 24 h, and 6/44 at 48 h. Only the preoperative-to-6 h McNemar comparison remained significant after Holm correction (adjusted *p* = 0.023). For TNF-alpha, only 24–48 h remained significant (adjusted *p* = 0.012). No IL-1alpha or IL-10 pair remained significant after correction despite borderline omnibus Q tests.

The proportion of readings was not flagged below the lower limit of quantification for each cytokine at each of the four timepoints, with an assayed subcohort, and denominator 44 participants per cell.

### 3.4. Assay Calibration and Quality Control

The standard-curve records were incomplete. Where available, curve fits were close to r^2^ = 0.99 (IL-10 plate 2, r^2^ = 0.9996; TNF-α plates 1 and 2, r^2^ = 0.9975 and 0.9924), whereas the IL-6 curve had r^2^ = 0.9591 and its 75 and 150 pg/mL standards gave a near-identical OD, indicating a plateau above approximately 37 pg/mL. The standard-curve records of the second IL-1α plate and the first IL-1β plate were corrupt or absent. Without duplicate precision, control samples, coefficients of variation, or acceptance criteria, no plate can be retrospectively certified as analytically valid.

### 3.5. Clinical Context and Sensitivity Analyses

In the same 44 participants, CRP, fibrinogen, and leukocytes increased after surgery while haemoglobin decreased (Table 6). These conventional markers document a postoperative acute-phase response but do not validate the below-range cytokine measurements. Descriptive arm-stratified OD summaries showed a similar nominal IL-6 pattern in the no-block and block groups (Supplementary Table S2); no between-arm inference was performed. The parent trial included 52 participants; cytokines were assayed in a selected 44-patient subcohort. Complete-case OD populations ranged from 16 to 39, whereas detectability flags were available for all 44 participants at all four timepoints.

## 4. Discussion

### 4.1. Principal Findings

The data directly support three findings about the dataset itself. First, most results were below the reportable range: two-thirds overall and 87.5% for IL-6. Second, the records needed to evaluate the assay performance were incomplete: samples were run in singlicate without control samples, coefficients of variation and acceptance criteria were unavailable, one of two plates was unusable for IL-1α and IL-1β, and the digital instrument export was lost. Third, the recovered OD values are reproducible as a transcription of the printed reports, which does not establish their analytical validity.

Two temporal findings are supported on the scales on which they were measured. IL-6 was detectable in more participants at 6 h than at baseline, and TNF-α detectability fell between 24 and 48 h. The within-participant OD varied over time for four analytes, most clearly for IL-6, and the IL-6 OD pattern was similar in participants with and without a block. These are statements about the assay signal and result flags, not about cytokine concentrations.

### 4.2. Observations Suggested but Not Confirmed

The IL-6 OD maximum at 24 h and the highest IL-6 detectability at 6 h are compatible with an early postoperative rise, but they cannot locate a peak or estimate its magnitude: the 6 h and 24 h OD values did not differ, most results were below range, and detection was inconsistent within participants. Because intervals were counted from the baseline sample, sample B also preceded the sixth postoperative hour. Published peak times after arthroplasty range from about 6 to 48 h [9,11,13,14], and the present data cannot contribute to that question.

IL-10 OD and detectability were numerically highest at 24 h. IL-10 is a counter-regulatory cytokine [20] (Figure 6), but temporal concurrence with IL-6 is compatible with several mechanisms and does not demonstrate counter-regulation. No within-participant IL-6/IL-10 association was tested, because such an analysis on signals mostly below the calibrated range would add apparent precision without restoring validity.

The IL-1α OD decline was confined to plate 1, which held the first 22 enrolled participants, whose samples had also been stored longest (565–614 days). Storage is unlikely to produce a within-participant decline by itself, because each participant’s four samples were stored for almost the same time and assayed together, but longer storage could lower the overall signal and detectability, and plate-level factors, including plate position, remain possible explanations. The IL-1β OD result was inconclusive (*n* = 16). IL-1β detectability was essentially fixed within participants, which points to a participant- or plate-level determinant rather than a temporal change. Above-range IL-1β results (above 100 pg/mL) were unexpected, because serum IL-1β did not change significantly after total hip replacement in a previous multiplex study [18]; the above-range IL-1α results are similarly unexplained. A non-specific signal is possible, but, without spike-recovery or dilution-linearity data, it cannot be evaluated.

### 4.3. Comparison with Published Evidence

The published series report postoperative circulating IL-6 concentrations well above the 4.69 pg/mL lower limit of the IL-6 kit used here: a peak of 399 ± 140 pg/mL 6 h after hip and knee replacement [9], a median peak of 82.3 pg/mL after uncemented total hip replacement [10], means of 33 and 41 pg/mL 6 h and one day after total hip replacement [18], and 23.5 pg/mL (IQR 18.3–27.9) 4 h after total hip arthroplasty under spinal anaesthesia with a femoral nerve block [25]. Absolute concentrations are platform-dependent, but all of these values exceed this limit several-fold. None of our participants received an NSAID or dexamethasone; dexamethasone is recommended in procedure-specific guidance [26,27] and has been associated with lower postoperative IL-6 after knee arthroplasty [28]. Preoperative concentrations, by contrast, have been reported below this limit (3.43 ± 2.44 pg/mL before knee arthroplasty [11]), so a censored baseline was expected. The predominance of below-range IL-6 results after surgery is therefore difficult to attribute to biology alone.

Serum TNF-α and IL-1β did not change significantly after total hip replacement in a multiplex study [18], cytokine concentrations differ between wound drainage and systemic blood [15,16,17], and serum IL-10 increased after arthroplasty in one series [21] but not in another [18]. The present TNF-α, IL-1, and IL-10 results neither confirm nor contradict this literature, because their analytical validity is uncertain.

Conventional acute-phase markers were recorded in the parent trial but were not used here to validate the cytokine measurements, and no within-participant IL-6–C-reactive protein analysis was attempted, because the IL-6 scale was not validated.

### 4.4. Conclusions Concerning Assay Performance

The discrepancy between the expected and the recovered IL-6 signal has several possible explanations. The analytical factors are supported by the documented calibration problems: the IL-6 standard curve had the poorest fit in the panel and flattened above approximately 37 pg/mL, and no IL-6 result was flagged above range, although several published series report postoperative concentrations above that level [9,10]. Pre-analytical degradation is a less likely explanation, because samples were processed immediately, stored at −80 °C for 13.5–20.2 months, and not thawed before the assay, conditions under which serum cytokines have been reported to remain stable [29]; it cannot be excluded for this kit. Matrix interference, dilution error, and data-recovery error are further possibilities. Without spike-recovery, dilution-linearity, duplicate, or control-sample data, the relative contribution of these factors cannot be determined.

These findings have practical implications for perioperative biomarker studies. The assay range should be compared with the concentrations expected for each analyte, procedure, and timepoint, and assay performance should be verified in representative perioperative samples, including spike recovery, dilution linearity, and duplicate precision, before a cohort is committed to it. Samples should be randomised across plates and plate positions, quality-control samples included on every plate, and digital exports, lot numbers, and acceptance criteria retained. Validated concentrations should then be analysed with methods for left-censored repeated measurements [21,22,23] rather than with the ranks of the below-range signal.

The reproducible contribution of this dataset is methodological. It shows that a perioperative ELISA dataset can yield highly significant within-participant OD differences while most results lie below the reportable range, and it provides an audit trail that keeps OD, printed result flags, and transcription reproducibility separate. It provides no basis for clinical decisions or biomarker thresholds. Because the samples could not be re-assayed, the perioperative cytokine trajectory in this setting can be established only by a new study with a fit-for-purpose assay and denser early sampling.

## 5. Strengths and Limitations

### 5.1. Strengths

The strengths the include transparent reconstruction of the available records, explicit quantification of censoring, paired analysis of detectability, correction across analytes, inclusion of the 44-versus-8 selection comparison, and presentation of conventional acute-phase markers from the same participants. The revised report separates reproducible assay findings from tentative biological interpretation.

### 5.2. Limitations

Assay range: Most IL-6 and IL-10 results were below the lowest calibrator, where concentration ordering was not established. OD significance therefore cannot establish cytokine concentration kinetics.

Data provenance: OD was reconstructed from scanned reports. Triangulation measures transcription reproducibility, not analytical validity, and the original digital export remains unavailable.

Pre-analytical and quality-control metadata: Tube type, processing interval, centrifugation, aliquoting, temperature, patient-level storage duration, freeze–thaw cycles, duplicate precision, kit lots, plate randomisation, blinding, acceptance criteria, and assay CVs were unavailable.

Structural missingness: IL-1alpha and IL-1beta analyses were each restricted to a single plate. Complete-case OD populations ranged from 16 to 39 and were not missing completely at random.

Selection: The assayed subcohort included 44/52 participants and all eight non-assayed participants were late-enrolled block recipients. Clinical differences between included and excluded patients limit generalisability.

Multiplicity and power: No cytokine was prespecified as primary. The analyses are post hoc and exploratory; multiplicity was controlled across analytes and within post hoc families, but small complete-case samples remain imprecise.

Timing: The exact reference point for the recorded postoperative intervals was not retained, preventing precise peak-timing inference.

Clinical heterogeneity: Regional block, ropivacaine, and rescue analgesia were present despite the absence of specified anti-inflammatory co-medication. Pooled summaries do not eliminate this heterogeneity.

Transferability: OD ranks are plate- and instrument-specific and cannot be compared with pg/mL results from other cohorts or used in meta-analysis.

## 6. Conclusions

In this secondary laboratory analysis of perioperative serum after total hip arthroplasty, most cytokine results were below the reportable range, one plate each for IL-1α and IL-1β was unusable, and the quality-control records needed to assess the assay performance were incomplete. The within-participant OD and detectability changed for some analytes, most clearly for IL-6, but these observations do not establish concentration kinetics, cytokine dominance, counter-regulation, or the absence of an IL-1 response. Because postoperative IL-6 results were far below the published postoperative concentrations and the samples could not be re-assayed, the defensible contribution of these data is methodological: the assay range and performance should be verified in representative perioperative samples, and complete digital and quality-control records retained, before biological interpretation is attempted.

## Figures and Tables

**Figure 1:**
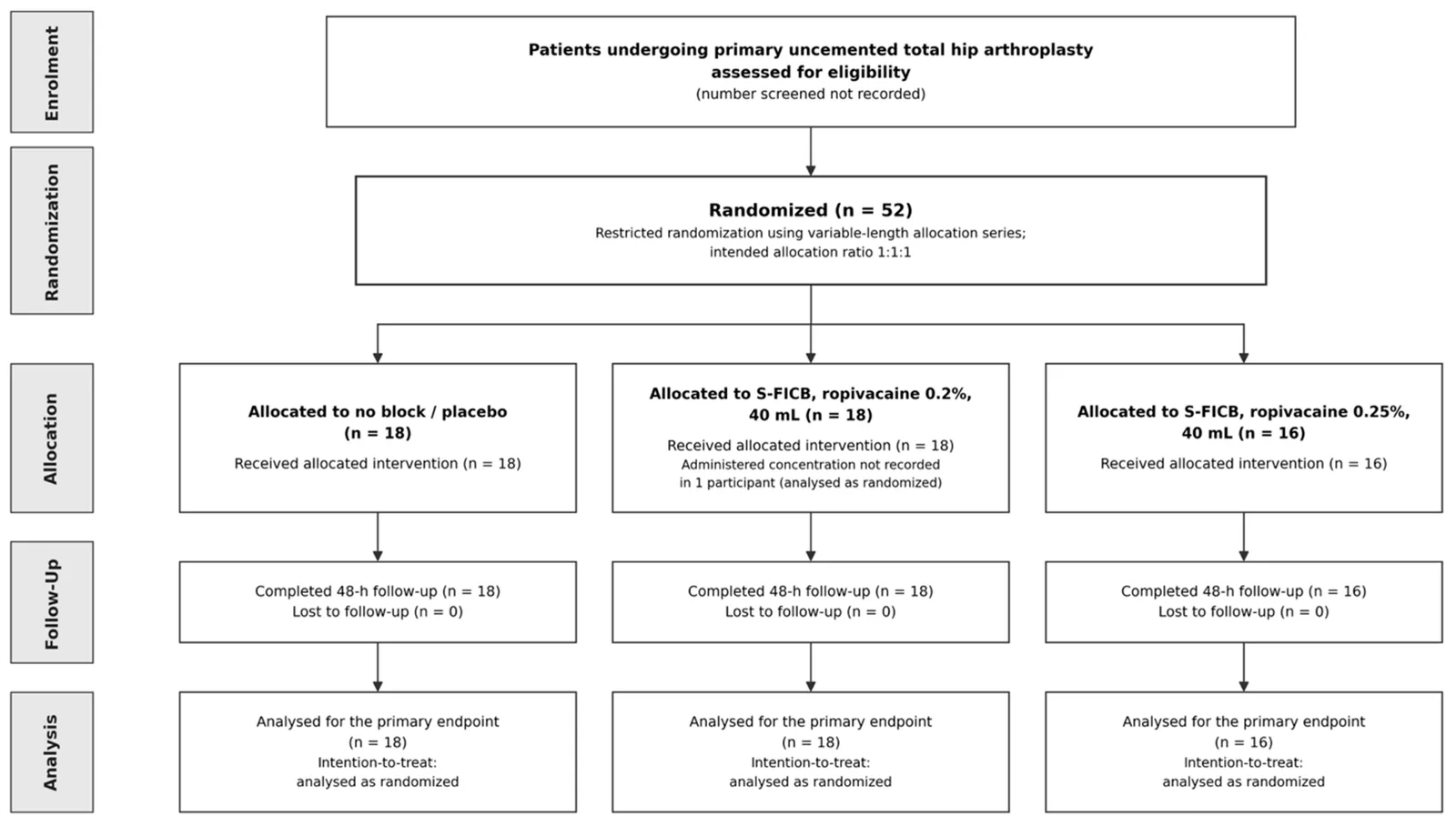
**Participant flow in the parent trial.** CONSORT flow diagram for the three-arm trial: no block (*n* = 18), S-FICB with 40 mL ropivacaine 0.2% (*n* = 18), and S-FICB with 40 mL ropivacaine 0.25% (*n* = 16). No sham or placebo procedure was performed. All participants completed 48 h follow-up and were analysed by intention to treat for the parent trial endpoint. Cytokines were assayed in 44 of the 52 participants.

**Figure 2:**
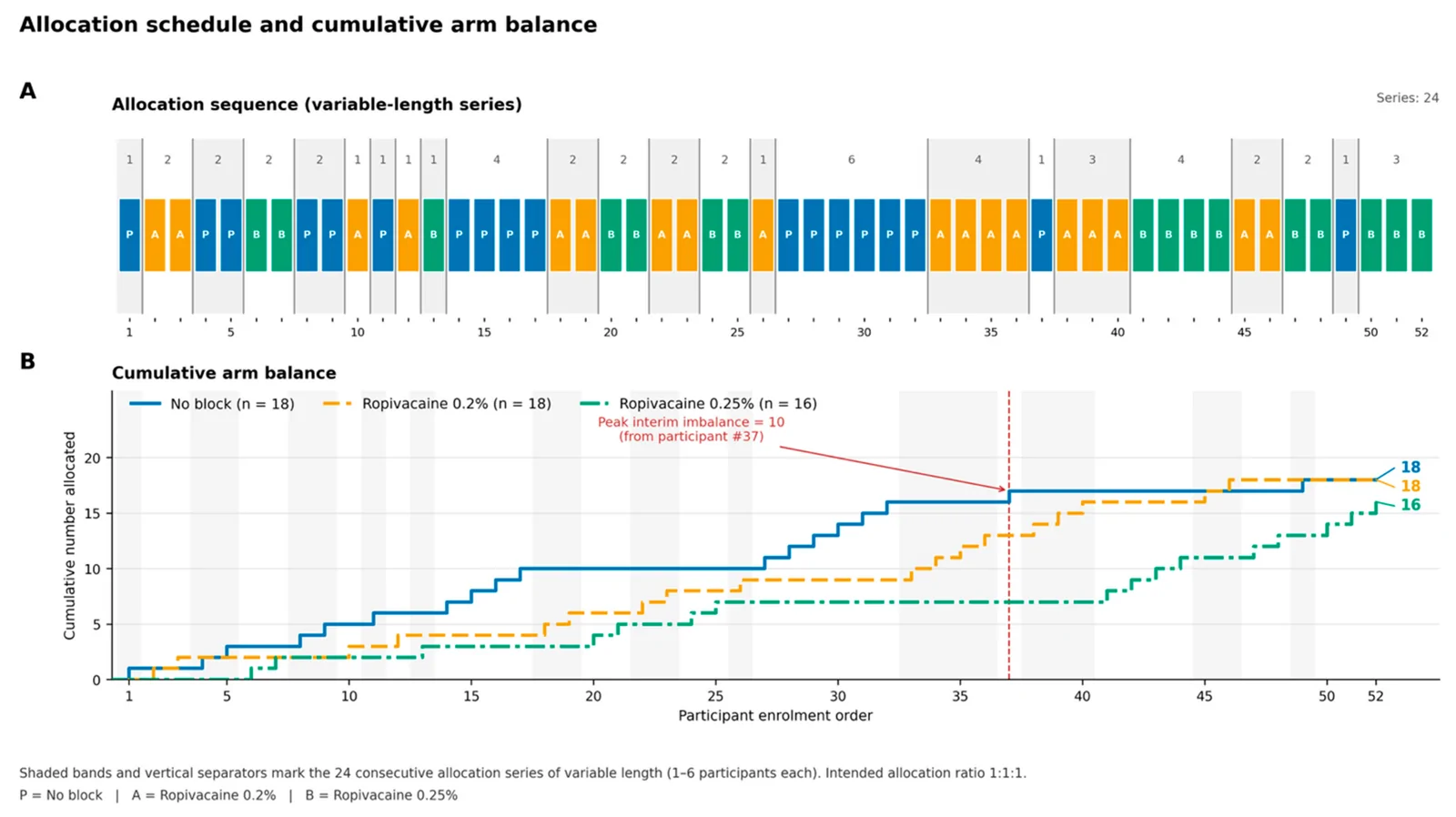
**Allocation schedule and cumulative arm balance.** (**A**) The realised allocation sequence across the 52 enrolled participants, arranged as 24 consecutive series of variable length (1–6 participants each); shaded bands and vertical separators mark the series boundaries and the figure above each band gives its length. P denotes no block, A ropivacaine 0.2% and B ropivacaine 0.25%. (**B**) Cumulative number allocated to each arm against enrolment order. Because allocation proceeded in per-arm series rather than within fixed balanced blocks, arm totals were not constrained to be equal at interim points; the maximum cumulative between-arm imbalance during recruitment was 10 participants, reached at participant 37, and the final allocation was 18:18:16.

**Figure 3:**
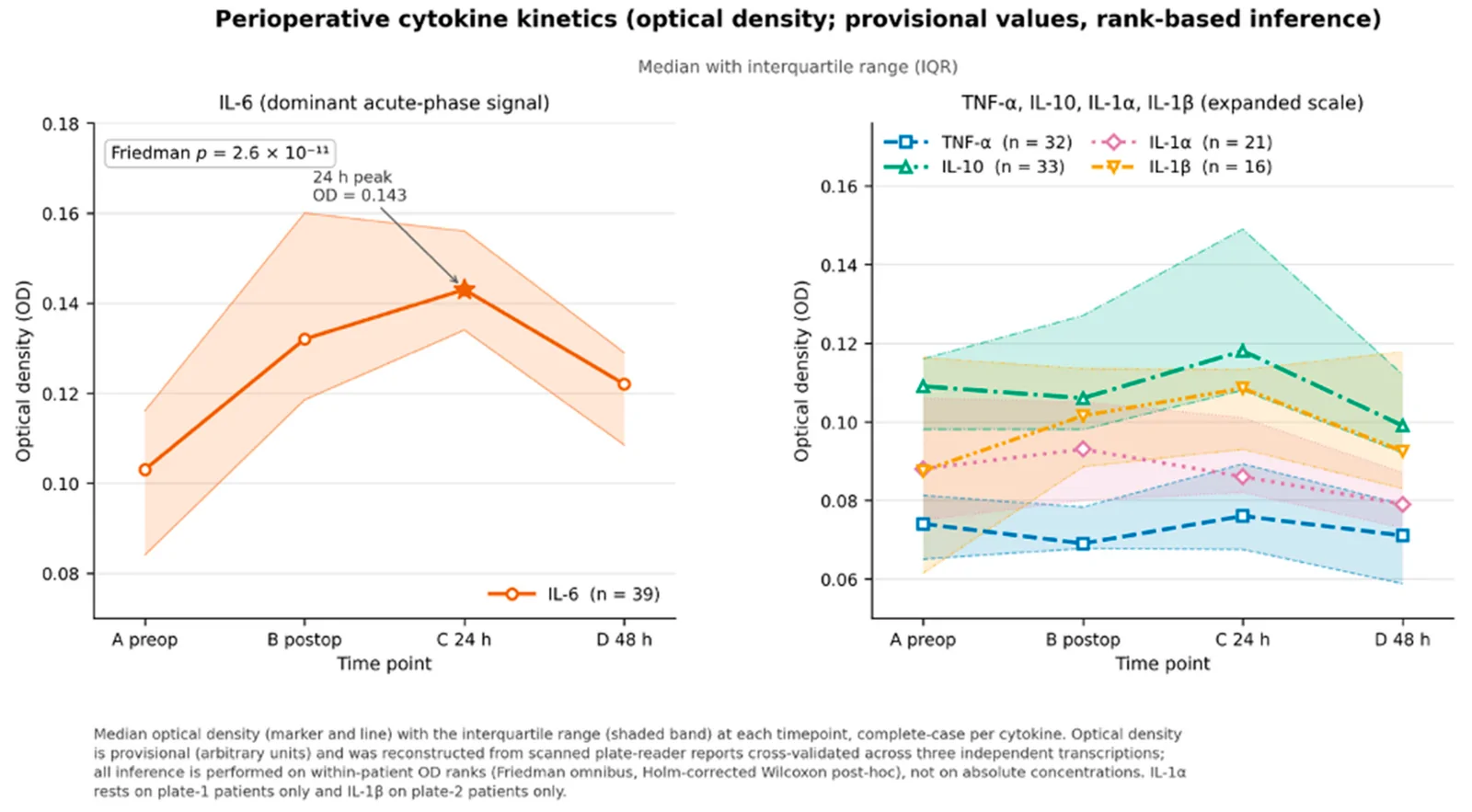
Perioperative optical-density trajectories of five serum cytokines.

**Figure 4:**
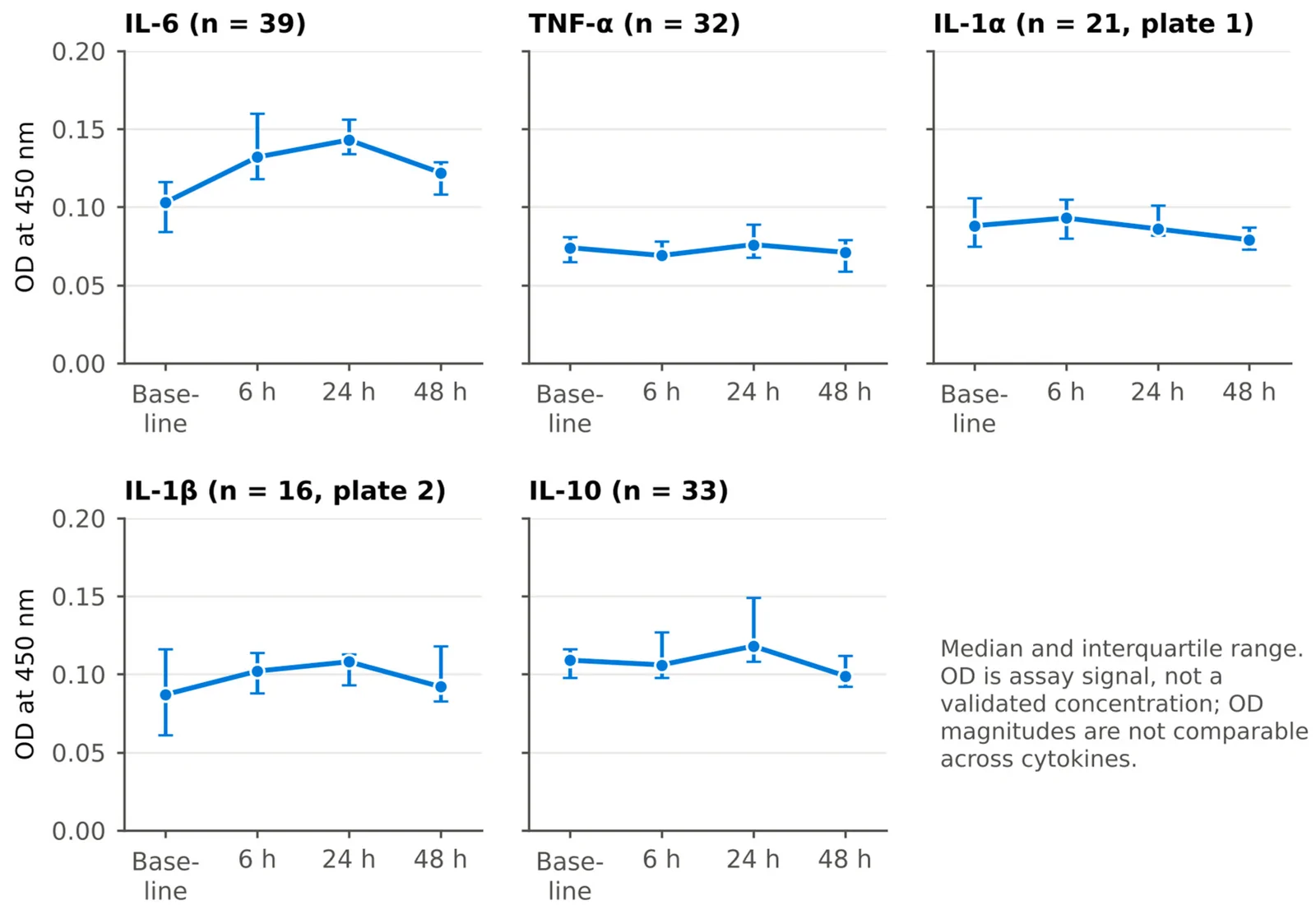
Recovered optical density by timepoint for five serum cytokines in participants with OD at all four timepoints. Points show medians’ and bars’ interquartile ranges; the vertical axis starts at zero and is identical in all panels. OD values are assay signal, not validated concentrations: most IL-6 and IL-10 results were below the reportable range, where OD may not preserve concentration order, and OD magnitudes cannot be compared across cytokines. IL-1α is restricted to plate 1 and IL-1β to plate 2. A, baseline sample before antibiotic prophylaxis; B, C, and D, 6, 24, and 48 h after the baseline sample.

**Figure 5:**
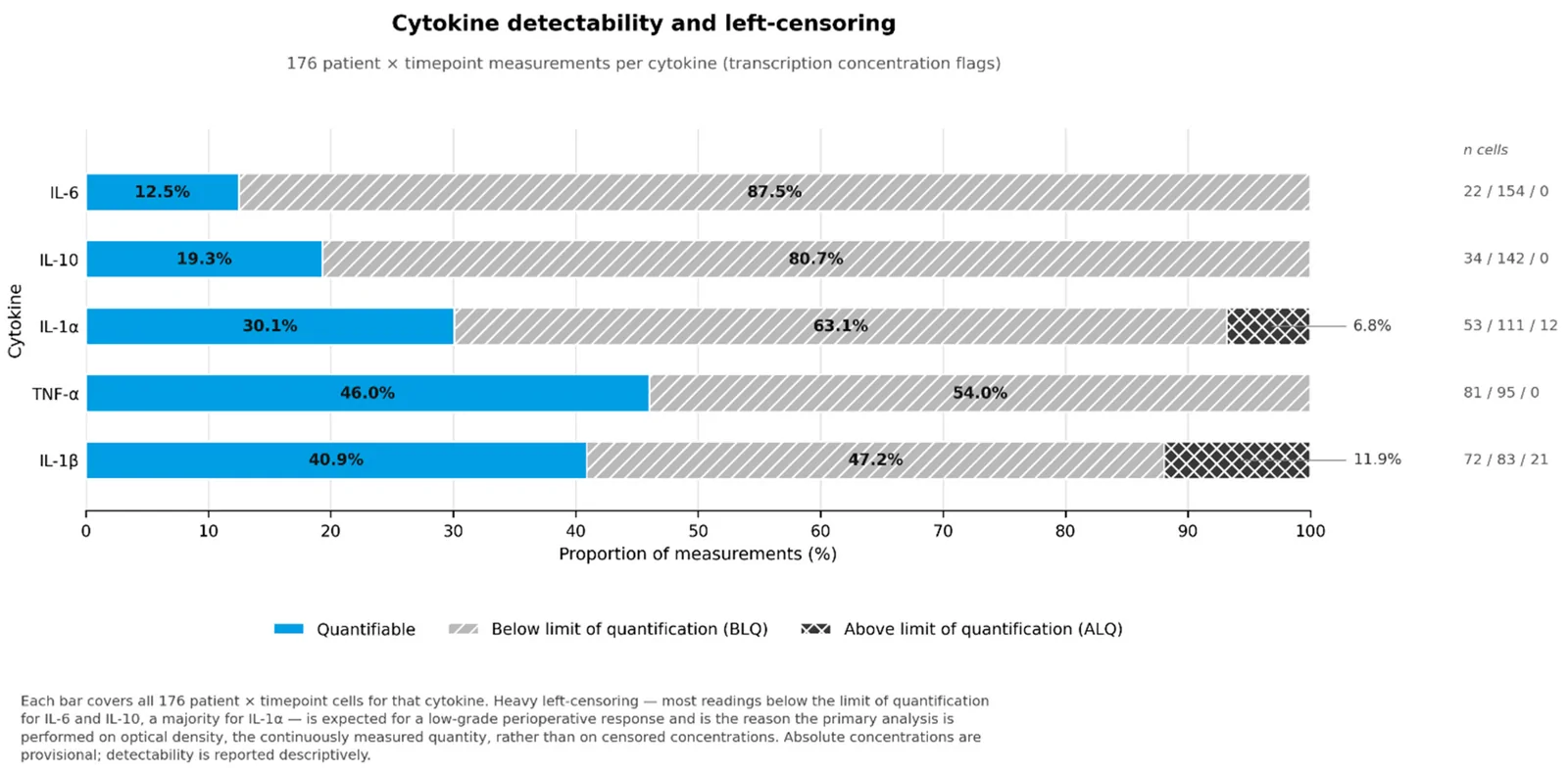
Detectability by cytokine and timepoint.

**Figure 6:**
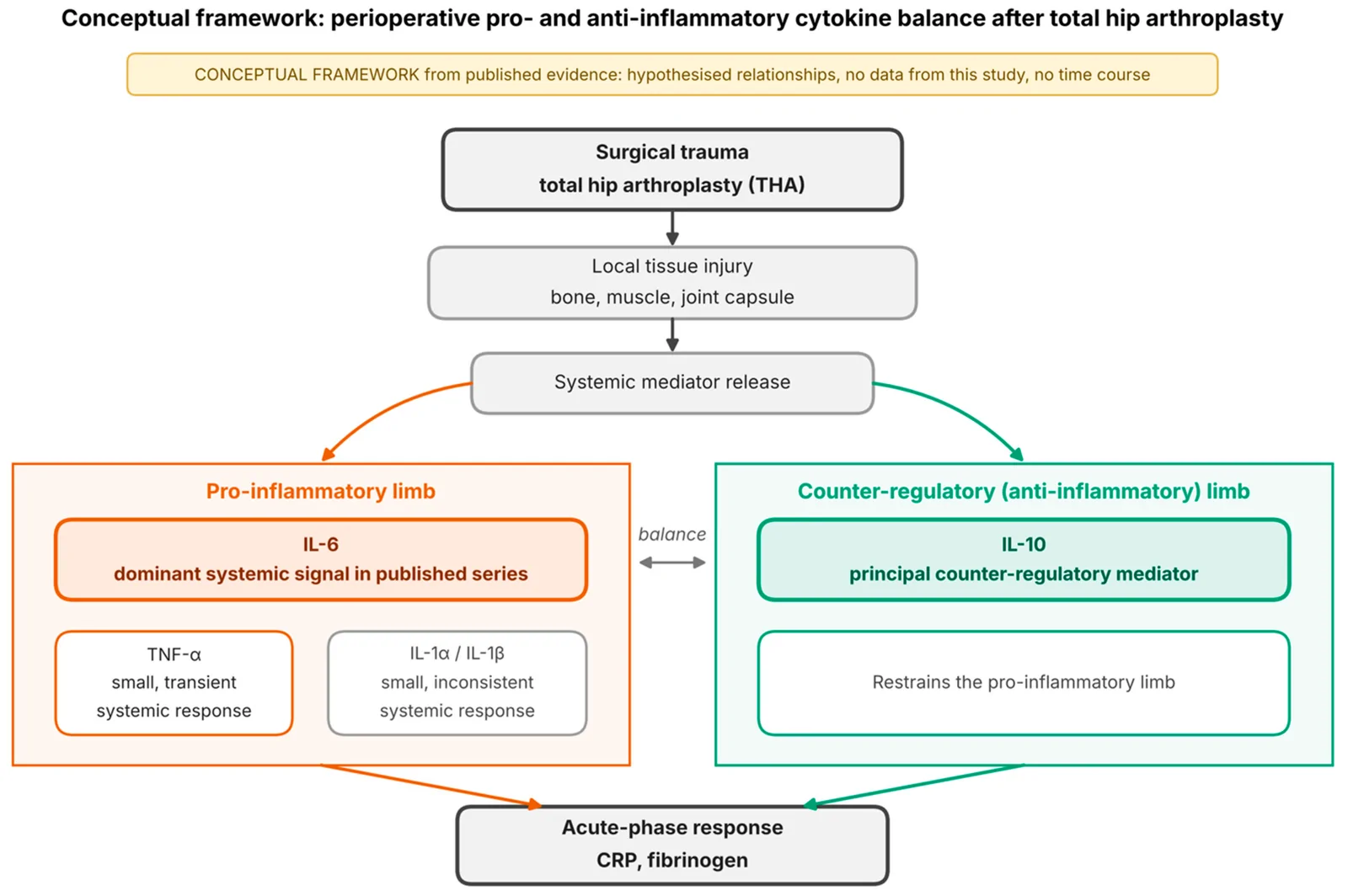
Conceptual framework of the perioperative cytokine response, based on published evidence. The pro-inflammatory limb (IL-6, the dominant systemic signal in published series; TNF-α and IL-1α/IL-1β, with smaller and less consistent systemic responses) and the counter-regulatory limb (IL-10) are shown converging on the hepatic acute-phase response (C-reactive protein, fibrinogen). Arrows denote hypothesised directions of influence, not estimated effects. The figure contains no data from the present study and shows no time course; the pathways depicted were not measured here, and these data cannot establish the timing of any cytokine peak.

**Table 1:** Cytokine assays, standard-curve ranges, and limits of quantification.

Cytokine	Kit Code	Standard-Curve Range (pg/mL)	LLOQ (pg/mL)
IL-6	EH0201	4.69–300	4.69
TNF-α	EH0302	15.6–1000	15.6
IL-1α	EF0184	3.9–250	3.9
IL-1β (high sensitivity)	EH018S-HS	1.56–100	1.56
IL-10	EH0173	7.81–500	7.81

All kits FineTest (Wuhan Fine Biotech, Wuhan, China). Absorbance read at 450 nm on a BIOMED LEDETECT 96 reader with MikroWin software. Each plate carried a seven-point standard curve (serial 1:2 dilutions) plus a blank; concentrations were derived by four-parameter logistic regression. LLOQ, lower limit of quantification. Ranges and limits are the manufacturer’s nominal values and were not independently established in this laboratory.

**Table 2:** Clinical and perioperative characteristics of assayed and non-assayed participants.

Characteristic	Assayed (*n* = 44)	Not Assayed (*n* = 8)	Standardised Difference
Age, years	67.0 [58.0–73.2]	64.0 [61.8–71.0]	−0.13
Female sex	24 (54.5)	7 (87.5)	−0.78
Body mass index, kg/m^2^	27.5 [25.1–30.5]	30.5 [28.9–32.9]	−0.93
ASA physical status III	4 (9.1)	2 (25.0)	−0.43
Operative duration, min	90.0 [70.0–101.2]	80.0 [70.0–90.0]	0.44
Total recorded blood loss, mL	1111 [850–1519]	1050 [800–1438]	0.21
Any transfusion	7 (15.9)	1 (12.5)	0.10
Received regional block	26 (59.1)	8 (100.0)	−1.18
Rescue morphine to 24 h, mg	10.0 [8.0–20.0]	8.0 [6.0–8.5]	0.62
Paracetamol/nefopam rescue doses, 24–48 h	5.0 [5.0–6.0]	4.5 [3.8–5.2]	0.69

**Table 3:** Perioperative cytokine trajectories: optical density at 450 nm, median [interquartile range], with within-patient nonparametric tests.

Cytokine	*n*	A: Preoperative	B: 6 h	C: 24 h	D: 48 h	Friedman *p* (Holm-Adjusted)	Pairs Significant After Holm Correction
IL-6	39	0.103 [0.084–0.116]	0.132 [0.118–0.160]	0.143 [0.134–0.156]	0.122 [0.108–0.129]	2.6 × 10^−11^ (1.3 × 10^−10^)	A–B, A–C, A–D, B–D, C–D
TNF-α	32	0.074 [0.065–0.081]	0.069 [0.068–0.078]	0.076 [0.068–0.089]	0.071 [0.059–0.079]	5.8 × 10^−4^ (0.0023)	C–D
IL-1α	21	0.088 [0.075–0.106]	0.093 [0.080–0.105]	0.086 [0.082–0.101]	0.079 [0.073–0.087]	1.0 × 10^−3^ (0.0023)	B–D, C–D
IL-1β	16	0.087 [0.061–0.116]	0.102 [0.088–0.114]	0.108 [0.093–0.113]	0.092 [0.083–0.118]	0.196 (0.196)	none
IL-10	33	0.109 [0.098–0.116]	0.106 [0.098–0.127]	0.118 [0.108–0.149]	0.099 [0.092–0.112]	6.6 × 10^−4^ (0.0023)	C–D

Values are optical density at 450 nm, median [IQR], in participants with OD at all four timepoints. Friedman *p*-values are shown unadjusted and, in parentheses, Holm-adjusted across the five analytes; pairwise Wilcoxon signed-rank comparisons were Holm-adjusted within analyte. IL-1α, plate 1 only; IL-1β, plate 2 only. Results below the calibrated range are assay-signal observations, not validated cytokine concentrations. A, baseline sample before antibiotic prophylaxis; B, C, and D, 6, 24, and 48 h after the baseline sample.

**Table 4:** Detectability across the whole study period, by cytokine (176 patient × timepoint cells per cytokine).

Cytokine	Numeric Result, *n* (%)	Flagged Below Range (BLQ), *n* (%)	Flagged Above Range (ALQ), *n* (%)	Detectable, *n* (%)
IL-6	22 (12.5)	154 (87.5)	0 (0)	22 (12.5)
TNF-α	81 (46.0)	95 (54.0)	0 (0)	81 (46.0)
IL-1α	53 (30.1)	111 (63.1)	12 (6.8)	65 (36.9)
IL-1β	72 (40.9)	83 (47.2)	21 (11.9)	93 (52.8)
IL-10	34 (19.3)	142 (80.7)	0 (0)	34 (19.3)
All analytes	262 (29.8)	585 (66.5)	33 (3.8)	295 (33.5)

Denominator is 44 participants × 4 timepoints = 176 results per cytokine, 880 in total. Categories are the flags printed by the reader software; a numeric result was not verified to lie within the manufacturer’s quantification range. Detectable = numeric or ALQ; ALQ results are detectable but not quantitatively interpretable. The manufacturer’s LLOQ and ULOQ were not independently validated in this laboratory.

**Table 5:** Detectability resolved by timepoint: readings not flagged below the limit of quantification per cytokine, assayed subcohort (*n* = 44).

Cytokine	A: Preoperative	B: 6 h	C: 24 h	D: 48 h	Total
IL-6	0/44	9/44	7/44	6/44	22
TNF-α	23/44	22/44	23/44	13/44	81
IL-1α	15/44	19/44	18/44	13/44	65
IL-1β	23/44	24/44	23/44	23/44	93
IL-10	5/44	8/44	12/44	9/44	34

Counts are readings that did not carry a below-limit flag and therefore include the above-limit readings. For IL-1α and IL-1β, the row totals (65 and 93) consequently exceed the quantifiable counts (53 and 72) by exactly the above-limit counts (12 and 21); for IL-6, TNF-α, and IL-10, which had no above-limit readings, the totals coincide.

**Table 6:** Conventional acute-phase and haematological markers in the 44 assayed participants.

Marker	Unit	*n* Complete	Preoperative	24 h	48 h	Friedman *p*
CRP	mg/dL	44	0.20 [0.20–0.50]	7.70 [5.67–9.10]	14.55 [10.78–19.23]	<0.001
Fibrinogen	mg/dL	44	329 [290–376]	450 [378–500]	500 [450–600]	<0.001
Leukocytes	/uL	44	6300 [6075–7525]	8050 [6900–9930]	7300 [6750–9575]	<0.001
Haemoglobin	g/dL	44	13.65 [13.17–14.30]	10.55 [9.80–11.72]	9.95 [9.28–11.15]	<0.001

Values are median [IQR] in participants with all three clinical laboratory timepoints. These results document the postoperative acute-phase response but do not validate below-range cytokine measurements.

## Data Availability

The original contributions presented in this study are included in the article. The reconciled optical-density dataset, with the printed result flags and the standard-curve values, is available from the corresponding author upon reasonable request.

## Supplementary Materials

The following supporting information can be downloaded at: https://www.mdpi.com/article/10.3390/jcm15207829/s1, Table S1. Clinical characteristics of the complete-case optical-density populations by analyte. Table S2. Descriptive optical-density sensitivity analysis by block exposure. Table S3. Exact paired McNemar comparisons of detectability. Figure S1. Participant and data flow, from the parent trial to the analyte-specific populations used for the exploratory OD analyses. Result flags were available for all 44 assayed participants at all four timepoints, whereas complete four-timepoint OD data were limited by scan quality and whole-plate loss. ALQ, flagged above the limit of quantification; BLQ, flagged below the limit of quantification; OD, optical density; S-FICB, supra-inguinal fascia iliaca compartment block.

## Abbreviations

4PL, four-parameter logistic; ALQ, above the limit of quantification; ASA, American Society of Anesthesiologists; BLQ, below the limit of quantification; COX-2, cyclo-oxygenase-2; CRP, C-reactive protein; ELISA, enzyme-linked immunosorbent assay; IL, interleukin; IQR, interquartile range; LLOQ, lower limit of quantification; NSAID, non-steroidal anti-inflammatory drug; OD, optical density; QC, quality control; THA, total hip arthroplasty; TNF-α, tumour necrosis factor-α; ULOQ, upper limit of quantification.
