# Supplementary material for: Assay Performance and Quantification Limits in Perioperative Serum Cytokine Measurement After Total Hip Arthroplasty: A Secondary Laboratory Analysis
Source: J Clin Med. 2026 Oct 10;15(20):7829. doi: 10.3390/jcm15207829 (PMC13705303; doi:10.3390/jcm15207829)
Supplement: Supplementary file 1 [file jcm-15-07829-s001.zip › jcm-4566727-supplementary.pdf]

Supplementary Materials

Table S1. Clinical characteristics of the complete-case optical-density populations by analyte.

Analyte	n	Age, years	Female, n (%)	Body mass index, kg/m²	ASA III, n (%)	Operative duration, min	No block, n (%)
IL-6	39	68.0 [57.5–73.5]	22 (56.4)	27.7 [25.6–30.8]	4 (10.3)	90.0 [70.0–100.0]	17 (43.6)
TNF-α	32	67.5 [57.0–74.0]	20 (62.5)	28.7 [25.2–31.4]	4 (12.5)	85.0 [70.0–100.0]	13 (40.6)
IL-1α	21	63.0 [59.0–72.0]	11 (52.4)	27.0 [25.1–30.0]	2 (9.5)	100.0 [70.0–110.0]	10 (47.6)
IL-1β	16	66.0 [55.0–72.5]	9 (56.3)	29.8 [26.4–31.8]	1 (6.3)	80.0 [70.0–90.0]	7 (43.8)
IL-10	33	67.0 [58.0–73.0]	20 (60.6)	27.3 [24.8–30.5]	3 (9.1)	90.0 [70.0–100.0]	16 (48.5)

Values are median [IQR] or n (%). Complete case means recoverable OD at all four timepoints. These subsets are determined partly by plate membership (IL-1α, plate 1 only; IL-1β, plate 2 only) and scan recovery and are not random samples. ASA, American Society of Anesthesiologists physical status.

Table S2. Descriptive optical-density sensitivity analysis by block exposure.

Analyte	Stratum	n	A: baseline	B: 6 h	C: 24 h	D: 48 h	Friedman p
IL-6	No block	17	0.109 [0.093–0.119]	0.131 [0.119–0.160]	0.143 [0.137–0.153]	0.115 [0.099–0.127]	1.4×10^{-5}
IL-6	Block	22	0.097 [0.083–0.113]	0.137 [0.118–0.159]	0.138 [0.132–0.157]	0.123 [0.109–0.129]	4.2×10^{-6}
TNF-α	No block	13	0.073 [0.063–0.077]	0.068 [0.063–0.075]	0.082 [0.070–0.090]	0.071 [0.061–0.079]	0.059
TNF-α	Block	19	0.075 [0.066–0.082]	0.073 [0.068–0.082]	0.075 [0.066–0.088]	0.069 [0.058–0.076]	0.0019
IL-1α	No block	10	0.096 [0.092–0.126]	0.102 [0.091–0.155]	0.101 [0.086–0.122]	0.084 [0.076–0.106]	0.015
IL-1α	Block	11	0.080 [0.068–0.086]	0.084 [0.080–0.093]	0.083 [0.077–0.087]	0.076 [0.071–0.081]	0.079
IL-1β	No block	7	0.106 [0.072–0.149]	0.111 [0.100–0.135]	0.112 [0.098–0.146]	0.109 [0.085–0.155]	0.60
IL-1β	Block	9	0.087 [0.062–0.113]	0.092 [0.086–0.108]	0.098 [0.093–0.109]	0.090 [0.081–0.099]	0.39
IL-10	No block	16	0.109 [0.097–0.117]	0.104 [0.098–0.123]	0.117 [0.107–0.148]	0.106 [0.093–0.115]	0.044

Analyte	Stratum	n	A: baseline	B: 6 h	C: 24 h	D: 48 h	Friedman p
IL-10	Block	17	0.107 [0.098–0.116]	0.113 [0.103–0.127]	0.118 [0.109–0.160]	0.098 [0.090–0.112]	0.021

Values are OD at 450 nm, median [IQR], in complete cases. Block = the two S-FICB arms combined (ropivacaine 0.2% and 0.25%). Friedman p-values are within-stratum and unadjusted; no between-arm test was performed. These exploratory summaries are not treatment-effect estimates and do not establish concentration kinetics below the calibrated range.

Table S3. Exact paired McNemar comparisons of detectability.

Analyte	Comparison	Lost / gained detection, n	Change, percentage points (95% CI)	Exact p	Holm-adjusted p
IL-6	A–B	0 / 9	20.5 (10.8 to 34.5)	0.0039	0.023
IL-6	A–C	0 / 7	15.9 (6.6 to 29.4)	0.016	0.078
IL-6	A–D	0 / 6	13.6 (4.5 to 26.7)	0.031	0.125
IL-6	B–C	5 / 3	−4.5 (−18.8 to 9.3)	0.727	1.00
IL-6	B–D	7 / 4	−6.8 (−22.5 to 8.7)	0.549	1.00
IL-6	C–D	2 / 1	−2.3 (−13.3 to 8.0)	1.00	1.00
TNF-α	A–B	5 / 4	−2.3 (−17.0 to 12.3)	1.00	1.00
TNF-α	A–C	5 / 5	0.0 (−15.2 to 15.2)	1.00	1.00
TNF-α	A–D	13 / 3	−22.7 (−39.4 to −5.4)	0.021	0.106
TNF-α	B–C	4 / 5	2.3 (−12.3 to 17.0)	1.00	1.00
TNF-α	B–D	11 / 2	−20.5 (−36.2 to −5.0)	0.022	0.106
TNF-α	C–D	10 / 0	−22.7 (−37.0 to −12.8)	0.0020	0.012
IL-1α	A–B	1 / 5	9.1 (−2.4 to 22.2)	0.219	0.875
IL-1α	A–C	2 / 5	6.8 (−6.0 to 20.5)	0.453	1.00
IL-1α	A–D	4 / 2	−4.5 (−17.6 to 7.8)	0.688	1.00
IL-1α	B–C	2 / 1	−2.3 (−13.3 to 8.0)	1.00	1.00
IL-1α	B–D	6 / 0	−13.6 (−26.7 to −4.5)	0.031	0.188
IL-1α	C–D	5 / 0	−11.4 (−24.0 to −2.4)	0.062	0.312
IL-1β	A–B	0 / 1	2.3 (−5.9 to 11.8)	1.00	1.00
IL-1β	A–C	0 / 0	0.0 (−8.0 to 8.0)	1.00	1.00
IL-1β	A–D	0 / 0	0.0 (−8.0 to 8.0)	1.00	1.00
IL-1β	B–C	1 / 0	−2.3 (−11.8 to 5.9)	1.00	1.00
IL-1β	B–D	1 / 0	−2.3 (−11.8 to 5.9)	1.00	1.00
IL-1β	C–D	0 / 0	0.0 (−8.0 to 8.0)	1.00	1.00
IL-10	A–B	0 / 3	6.8 (−1.8 to 18.2)	0.250	1.00
IL-10	A–C	1 / 8	15.9 (3.2 to 30.3)	0.039	0.234

Analyte	Comparison	Lost / gained detection, n	Change, percentage points (95% CI)	Exact p	Holm-adjusted p
IL-10	A–D	0 / 4	9.1 (0.3 to 21.2)	0.125	0.625
IL-10	B–C	3 / 7	9.1 (−5.7 to 24.2)	0.344	1.00
IL-10	B–D	2 / 3	2.3 (−9.7 to 14.6)	1.00	1.00
IL-10	C–D	5 / 2	−6.8 (−20.5 to 6.0)	0.453	1.00

A, baseline; B, 6 h; C, 24 h; D, 48 h. Lost = detectable at the earlier timepoint but not the later one; gained = the reverse. Change = later minus earlier proportion detectable (n = 44). CIs are asymptotic score intervals (Tango) and are not adjusted for multiplicity; with few discordant pairs they can exclude zero when the exact test is not significant. Holm adjustment was applied to the six comparisons within each analyte. Comparisons for IL-1α, IL-1β and IL-10 are shown for completeness; their Cochran's Q was not significant after adjustment across analytes. Detectable means not flagged below range; above-range results are detectable but not quantitatively interpretable.

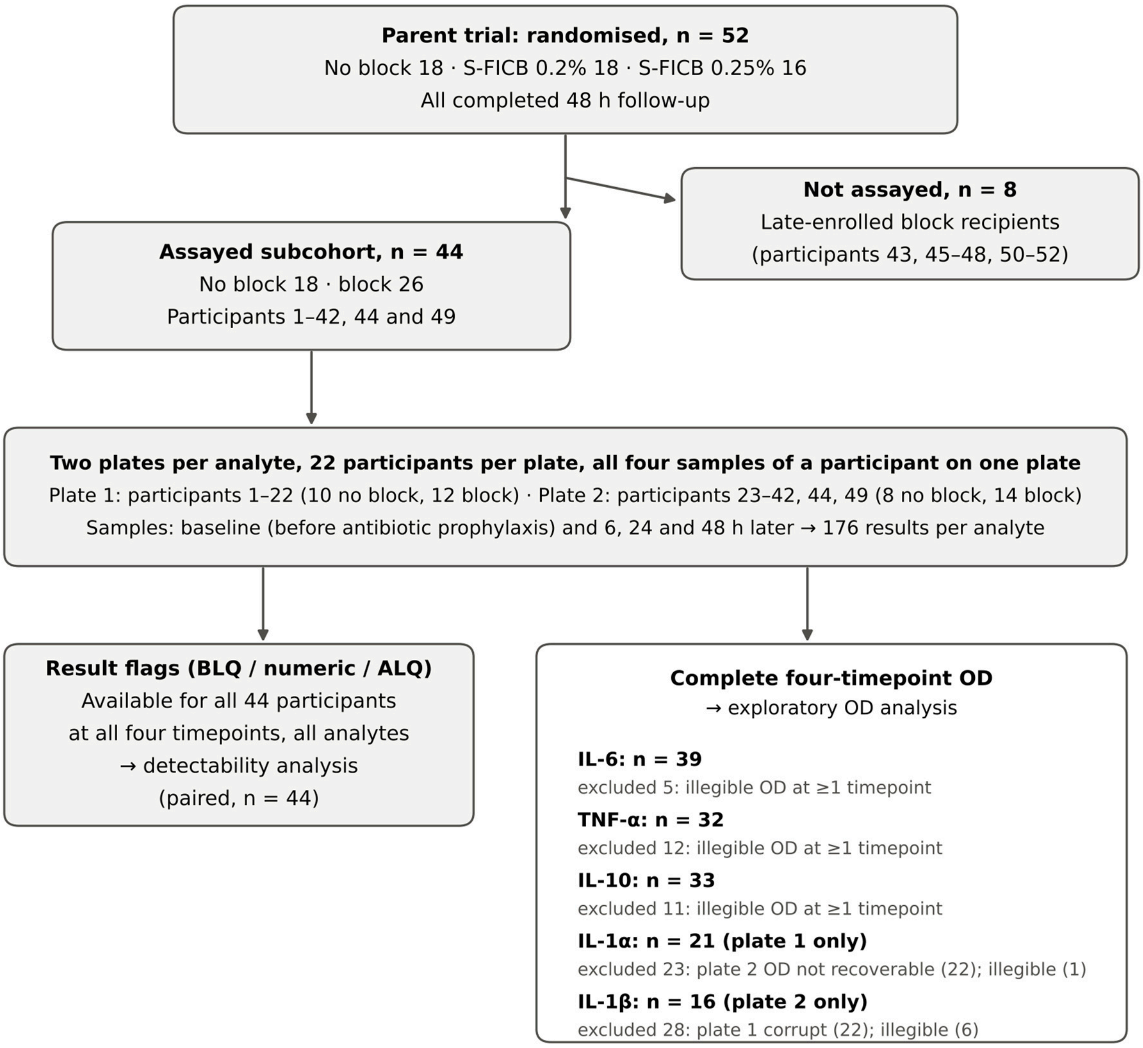

Figure S1. Participant and data flow, from the parent trial to the analyte-specific populations used for the exploratory OD analyses. Result flags were available for all 44 assayed participants at all four timepoints, whereas complete four-timepoint OD data were limited by scan quality and whole-plate loss. ALQ, flagged

above the limit of quantification; BLQ, flagged below the limit of quantification; OD, optical density; S-FICB, supra-inguinal fascia iliaca compartment block.
